# Consensus document for the diagnosis of prosthetic joint infections: a joint paper by the EANM, EBJIS, and ESR (with ESCMID endorsement)

**DOI:** 10.1007/s00259-019-4263-9

**Published:** 2019-01-26

**Authors:** Alberto Signore, Luca Maria Sconfienza, Olivier Borens, Andor W. J. M. Glaudemans, Victor Cassar-Pullicino, Andrej Trampuz, Heinz Winkler, Olivier Gheysens, Filip M. H. M. Vanhoenacker, Nicola Petrosillo, Paul C. Jutte

**Affiliations:** 1 Nuclear Medicine Unit, Department of Medical-Surgical Sciences and of Translational Medicine, Faculty of Medicine and Psychology, “Sapienza” University of Rome, Rome, Italy; 2 Unit of Diagnostic and Interventional Radiology, IRCCS Istituto Ortopedico Galeazzi, Milan, Italy; 3 Department of Biomedical Sciences for Health, Università degli Studi di Milano, Milan, Italy; 4 Division of Orthopaedic Surgery and Traumatology, Lausanne University Hospital, Lausanne, Switzerland; 5 Department of Nuclear Medicine and Molecular Imaging, University Medical Center Groningen, University of Groningen, Groningen, The Netherlands; 6 Department of Diagnostic Imaging, The Robert Jones and Agnes Hunt Orthopaedic and District Hospital – Oswestry, Shropshire, UK; 7 Department of Internal Medicine, University Hospital “Charitè”, Berlin, Germany; 8 Osteitis-Centre, Privatklinik Döbling, Vienna, Austria; 9 Department of Nuclear Medicine, University Hospital Leuven, Leuven, Belgium; 10 Department of Radiology, University Hospital Antwerp, AZ Sint-Maarten Duffel-Mechelen, and University of Ghent, Edegem, Belgium; 11 Division of Infective Diseases, National Institute for Infective Diseases “L. Spallanzani”, Rome, Italy; 12 Department of Orthopaedic Surgery, University of Groningen, University Medical Center Groningen, Groningen, The Netherlands

**Keywords:** Infection diagnosis, Prosthetic joint infection, Imaging, Guideline

## Abstract

**Background:**

For the diagnosis of prosthetic joint infection, real evidence-based guidelines to aid clinicians in choosing the most accurate diagnostic strategy are lacking.

**Aim and Methods:**

To address this need, we performed a multidisciplinary systematic review of relevant nuclear medicine, radiological, orthopaedic, infectious, and microbiological literature to define the diagnostic accuracy of each diagnostic technique and to address and provide evidence-based answers on uniform statements for each topic that was found to be important to develop a commonly agreed upon diagnostic flowchart.

**Results and Conclusion:**

The approach used to prepare this set of multidisciplinary guidelines was to define statements of interest and follow the procedure indicated by the Oxford Centre for Evidence-based Medicine (OCEBM).

**Electronic supplementary material:**

The online version of this article (10.1007/s00259-019-4263-9) contains supplementary material, which is available to authorized users.

## Preamble

The European Association of Nuclear Medicine (EANM) is a professional nonprofit medical association that facilitates communication worldwide between individuals pursuing clinical and research excellence in nuclear medicine. The EANM was founded in 1985. EANM members are physicians, technologists, and scientists specializing in the research and practice of nuclear medicine.

The EANM will periodically define new guidelines for nuclear medicine practice to help advance the science of nuclear medicine and to improve the quality of service to patients throughout the world. Existing practice guidelines will be reviewed for revision or renewal, as appropriate, on their fifth anniversary or sooner, if indicated.

Each practice guideline, representing a policy statement by the EANM, has undergone a thorough consensus process in which it has been subjected to extensive review. The EANM recognizes that the safe and effective use of diagnostic nuclear medicine imaging requires specific training, skills, and techniques, as described in each document. Reproduction or modification of the published practice guideline by those entities not providing these services is not authorized.

These guidelines are an educational tool designed to assist practitioners in providing appropriate care for patients. They are not inflexible rules or requirements of practice and are not intended, nor should they be used, to establish a legal standard of care. For these reasons and those set forth below, the ESR, EBJIS, ESCMID, and the EANM suggest caution against the use of these guidelines in litigation in which the clinical decisions of a practitioner are called into question.

The ultimate judgment regarding the propriety of any specific procedure or course of action must be made by the physician or medical physicist in light of all the circumstances presented. Thus, there is no implication that an approach differing from the guidelines, standing alone, is below the standard of care. To the contrary, a conscientious practitioner may responsibly adopt a course of action different from that set forth in the guidelines when, in the reasonable judgment of the practitioner, such course of action is indicated by the condition of the patient, limitations of available resources, or advances in knowledge or technology subsequent to publication of the guidelines.

The practice of medicine includes both the art and the science of the prevention, diagnosis, alleviation, and treatment of disease. The variety and complexity of human conditions makes it impossible to always reach the most appropriate diagnosis or to predict with certainty a particular response to treatment. Therefore, it should be recognized that adherence to these guidelines will not ensure an accurate diagnosis or a successful outcome. All that should be expected is that the practitioner will follow a reasonable course of action based on current knowledge, available resources, and the needs of the patient, to deliver effective and safe medical care. The sole purpose of these guidelines is to assist practitioners in achieving this objective.

## Introduction

Because of increased life expectancy, the number of joint prosthesis replacements continues to grow at a significant rate, with several millions prosthetic joints implanted each year worldwide [1]. Most of the time this surgery results in better joint function and pain relief, and the procedure itself is safe and cost-effective [2]. However, prosthetic joint infection (PJI) is a complication that can occur, with an incidence ranging from 2.0% to 2.4% for primary interventions [3], but increasing to as high as 20% for revision procedures [4], leading to a true economic burden, since costs are very high when an infection develops [3]. PJI is a serious condition that may lead to repeated surgical interventions, prolonged hospitalization, high costs, and significant morbidity, although low mortality.

PJIs are usually classified in relation to the time of onset after surgery: (i) early, within the first 3 months after surgery, (ii) delayed (between 3 months and 2 years after surgery), and (iii) late (more than 2 years after surgery) [5]. Microorganisms may reach the prosthesis at the time of implantation or later by haematogenous spread [6, 7].

Early infection is often easy to recognize by acute pain in the wound area, redness, swelling, wound leakage, heat, and fever. Late infection is often caused by haematogenous spread, with symptoms from both the affected joint and the primary infection site. The main diagnostic problem is the delayed, often low-grade infection with mostly nonspecific symptoms. In all PJIs, a delay in starting an appropriate antibiotic regimen and surgical treatment has an important impact on the chance of saving the prosthesis and joint function. Therefore, early diagnosis is of utmost importance.

There is no single routine test available that can diagnose PJI with sufficient accuracy. Cultures, biopsies, serum inflammatory markers, and imaging techniques all have their pros and cons. C-reactive protein (CRP), erythrocyte sedimentation rate (ESR), and leukocyte count are not sensitive or specific enough to detect or exclude a PJI. Joint aspiration itself involves a risk of infection, and its sensitivity is highly variable. In general, a combination of clinical, laboratory, microbiological, and imaging tests are performed based on personal experience, techniques available in the institute, and cost aspects. Indeed, most signs and symptoms that might indicate the presence of a PJI may be simply related to an aseptic loosening of the prosthesis or to a soft tissue infection. Since loosening, soft tissue infection without osteomyelitis, and PJI require different treatment strategies (NSAIDs, antibiotic therapy, or surgery for explant and re-implant), the correct differential diagnosis among these clinical situations is crucial.

The development of biofilms plays a strategic role in the pathogenesis of PJI. Microorganisms adhere to the implant and form a biofilm, within which they are protected from the host immune system and most antibiotics [7, 8]. The most frequent etiologic agents are staphylococci, accounting for more than 50% of PJIs [8]. *Staphylococcus aureus* is most commonly isolated in early infection, whereas coagulase-negative staphylococci are more frequent in late infection [8].

Other commonly isolated organisms in late infections are streptococci (9–10%), enterococci (3–7%), and anaerobes (2–4%) [7]. Gram-negative bacteria, mostly *Pseudomonas aeruginosa*, *Enterobacter* spp., and *Proteus* spp., even if relatively uncommon agents, have an important clinical impact because of the difficulty in treating them [9, 10]. Overall, about 20% of PJIs are polymicrobial and 7–11% are culture-negative [11, 12]. Unusual pathogens such as *Candida* spp., *Brucella* spp., and mycobacteria have also been reported [13].

Other commonly isolated organisms in late infections are streptococci (9–10%), enterococci (3–7%), and anaerobes (2–4%) [7]. Gram-negative bacteria, mostly *Pseudomonas aeruginosa*, *Enterobacter* spp., and *Proteus* spp., even if relatively uncommon agents, have an important clinical impact because of the difficulty in treating them [9, 10]. Overall, about 20% of PJIs are polymicrobial and 7–11% are culture-negative [11, 12]. Unusual pathogens such as *Candida* spp., *Brucella* spp., and mycobacteria have also been reported [13].

There are several papers available with recommendations for diagnosing PJI, all with limitations: not focusing strictly on PJI, not focused on a diagnostic flow chart, not including nuclear medicine, based only on expert opinion and/or local consensus meetings, or not up to date (not analysing the currently available diagnostic techniques). Therefore, multidisciplinary evidence-based guidelines are needed, including the most relevant imaging techniques to support the most accurate diagnostic strategy. To achieve this goal, nuclear medicine physicians organized a pre-congress meeting with infectious disease specialists, radiologists, and orthopaedic surgeons on the occasion of the 20th congress of the European Association of Nuclear Medicine (EANM), where each professional discipline explained their points of view, and together they drafted a possible diagnostic flowchart for PJI [14]. As this was still only expert opinion, the aim now becomes to define a real evidence-based diagnostic flowchart for PJI by performing a thorough systematic review of the relevant literature in the areas of nuclear medicine, radiology, infectious diseases, and microbiology to define the diagnostic accuracy of each technique and to address and provide evidence-based answers on uniform statements for each topic found to be important for developing a diagnostic flowchart.

## Methods

### Working group

After several joint symposia and reading of several available guidelines based only on expert opinion, we recognised that a multidisciplinary evidence-based guideline for diagnosing peripheral bone infections was needed. This joint society project started in 2015, and a working group was created with delegates from four European societies: the European Association of Nuclear Medicine (EANM), the European Society of Radiology (ESR), the European Bone and Joint Infection Society (EBJIS), and the European Society of Clinical Microbiology and Infectious Diseases (ESCMID). The delegates first met in Vienna (November 2015) to define the statements and after that in Rome (February 2016) to jointly define the revised and final statements, based on the literature evidence that had first been circulated among all participants. Finally, all delegates approved the final version of each statement.

### Statements

Uniform statements were addressed for each topic, with the aim of positioning all diagnostic procedures in a commonly agreed upon and evidence-based diagnostic flowchart. Each consensus statement is followed by comments derived from analysis of the relevant literature.

### Literature search

An extensive literature search of the PubMed/Medline and Scopus databases was conducted (for the period from 1 January 2000 up to December 2015) for each statement and for overall diagnostic accuracy of each diagnostic technique. Search terms were defined in agreement with all delegates from the four participating societies. Inclusion of the papers per statement was based on a PICO (Population/problem – Intervention/indicator – Comparator – Outcome) question to search for evidence after converting the PICO question into a search strategy. This strategy is described in detail by the Oxford Centre for Evidence-based Medicine (OCEBM) [15]. Case reports, reviews, and papers with fewer than 10 patients were excluded, as well as non-English-language papers. Systematic reviews were included. A cross-search with references included in the retrieved articles was also performed to look for further evidence. Based on expert experience, if some known paper was not retrieved by the search, it was added for competence.

### Method for scoring the papers

All included papers per statement were thoroughly read and analysed, and a “level of evidence” was provided in consensus with all delegates for each paper according to the documents for levels of evidence provided by the OCEBM [16]. Finally, putting all levels of evidence for all included papers together, a final level of evidence was provided, again in agreement among all delegates, for each statement.

### Current diagnostic approach for PJI

The diagnostic approach in patients with suspected PJI is extremely variable from centre to centre, depending on local experience, technological equipment, and adherence to available guidelines.

However, thus far there are no published “evidence-based” guidelines to guide the diagnostic flow of PJI. Clinical examination and laboratory tests, together with a planar x-ray film, are the first steps. These are followed by biopsies, or joint aspiration with microbiological analysis or advanced imaging modalities such as radiological and nuclear medicine examinations. These are explained in more detail in Appendix 1 [17–47], and a statement on the concerns regarding the use of ionizing radiation is described in Appendix 2.

## Consensus statements

All PICOs performed for the statements and the list of publications selected for the level of evidence are mentioned in Appendix 3.

**1. PJI should be suspected when one or more of the following symptoms and signs are present: otherwise unexplained pain and/or fever, redness, swelling, scar inflammation, and movement limitations. These symptoms are (especially in the chronic phase) not specific and require other investigations.**

Level of evidence: 4.

The main clinical signs of early PJI are persistent local pain, erythema, swelling, wound healing disturbance (leakage), and fever. In delayed and chronic infection occurring many years after prosthesis insertion, clinical signs may be absent; when present, they are represented by persistent or increasing joint pain and a loose prosthesis.

Symptoms are not specific for infection, and it is not easy to distinguish PJI from aseptic loosening by clinical history and physical examination alone. As a consequence, patients with one or more of the above-mentioned signs and symptoms should undergo further investigation for diagnosing or excluding the presence of infection [48–54].

**2. Sinus tract and purulent discharge are clear signs of prosthetic joint infection.**

Level of evidence: 5.

There are no papers published on this specific issue; rather, this is a general belief based on the logical assumption that, given the natural colonization of the skin, these bacteria will certainly colonize the exposed implant. However, no literature was found providing evidence that a sinus tract with purulent discharge is a sign of PJI. There is no evidence that microbiological analysis of tissue or fluid from sinus tracts is reliable for diagnosis.

**3. CRP and ESR should always be performed in patients with suspected prosthetic joint infection. A normal value does not rule out PJI.**

Level of evidence: 2.

Patients presenting with clinical signs of prosthetic joint infection (i.e. fever, unexplained pain at the site of arthroplasty, prosthetic loosening, or a sinus tract) should be subjected to screening for inflammatory markers in serum. C-reactive protein (CRP) and erythrocyte sedimentation rate (ESR) are good preliminary tests. Screening of these markers can be performed rapidly, inexpensively, and with minimal inconvenience. Although there is considerable variation in sensitivity (SE) and specificity (SP) for both CRP (SE 21–100%, SP 20–96%) and ESR (SE 58–97%, SP 33–90.9%), in general they show good diagnostic value, and are of relevance particularly for therapy follow-up. A threshold of 10 mg/l for CRP and 30 mm/h for ESR of is recommended for diagnostic purposes. One level 1 study and 32 level 2 studies addressed the diagnostic efficacy of CRP and ESR in serum [19, 55–82]. However, when testing for CRP and ESR, one should keep in mind that both markers can be influenced by numerous factors (i.e. neoplastic and inflammatory conditions, age, and technical details). Furthermore, CRP is produced in the liver, and especially in low-grade chronic PJI, levels may not be elevated. Therefore, a careful history and physical examination are mandatory. Several studies suggest using the combination of ESR and CRP [56, 59, 83]. In any case, if one of these markers is above the threshold, further diagnostic tests should be performed. Low CRP and ESR do not rule out PJI.

**4. In the case of fever, blood cultures should always be performed in patients suspected of having prosthetic joint infection in order to identify the causative bacteria.**

Level of evidence: 5.

Microorganisms may reach the prosthesis at the time of implantation or later by haematogenous spread [6, 7].

Haematogenous seeding can occur at any time after joint implantation. The main sources of bacteraemia are skin, respiratory tract, dental procedures, and urinary tract infection [7]. Haematogenous spread of *S. aureus* can also occur in patients without a detectable primary focus [84].

No studies specifically assessed the role of blood cultures in diagnosing PJI. However, in consideration of the frequent haematogenous origin of PJI, it is recommended that blood cultures be performed in patients with suspected PJI who have fever.

**5. Conventional radiography is the first imaging modality to perform in patients with suspicion of PJI for diagnosis and follow-up.**

Level of evidence: 2.

Conventional radiography is regularly used to evaluate joint prostheses after implantation and follow-up, as these procedures are able to detect any potential abnormality involving both the implant and the surrounding bone. For this reason, conventional radiography should always be performed. Regarding PJI, conventional radiography often yields normal results or may detect nonspecific signs of soft tissue swelling. Serial plain radiography has been reported to have sensitivity of 14% and specificity of 70% in detecting implant-associated infections. Radiographic signs that may reveal PJI with high specificity are gas formation and active, immature periostitis. Radiographic signs with low specificity include soft tissue swelling, periprosthetic lucency, and component loosening. However, differentiation between septic and aseptic periprosthetic lucency and component loosening is almost impossible on conventional radiography. Also, these signs are visible only when almost 30% of the bone mass has been lost; thus, 50% of radiographs remain normal despite the presence of infection [85–87].

**6. Ultrasound can detect complications around the prosthesis, but the capacity for detecting infection is controversial.**

Level of evidence: 2.

Van Holsbeeck et al. reported 100% sensitivity and 74% specificity for infection when the a capsule-to-bone distance was >4 mm, while 100% specificity was observed when the capsule-to-bone distance was >3.2 mm and extracapsular fluid was found in the hip [88]. These findings were questioned by Weybright et al. [89], who reported that anterior distension of the hip capsule was not predictive of infection.

**7. Imaging may be useful for guiding joint aspiration or periprosthetic tissue biopsy.**

Level of evidence: 2.

One of the challenges of correct aspiration or biopsy in and around the implant is placing the needle exactly in the area of interest. Open aspiration and biopsy can be done in a surgical theatre, but this approach is highly invasive. Fluoroscopy can be used as a guide, but it does not correctly show soft tissues and fluids, and it uses ionizing radiation. US can be used to guide the needle in the joint space and in the soft tissue around the implant without the use of ionizing radiation. Eisler et al. reported 7% sensitivity and 66% specificity for ultrasound-guided capsular biopsy, while fluid aspiration was always falsely sterile [90]. Battaglia et al. demonstrated 69% sensitivity, 94% specificity, and 83% accuracy for ultrasound-guided periprosthetic joint aspiration [91]. CT-guided aspiration has been reported to have 70% sensitivity, 100% specificity, and 84% accuracy for diagnosis of infection [92].

**8. Leukocyte count and differential in synovial fluid has high diagnostic accuracy in detecting PJI.**

Level of evidence: 2.

Patients with abnormal CRP and/or ESR should undergo aspiration of the hip or knee. We recommend that aspiration fluid be tested for white blood cell count (WBC) and percentage of neutrophils. Our systematic review of the literature showed high sensitivity and specificity for both tests. Synovial white blood cell count had sensitivity of 36–100% and specificity of 80–99%. Differential count had sensitivity of 84–100% and specificity of 80–99%. A threshold for white blood cell count of >3000 cells/μl seems justified based on the studies found (the thresholds from various reports vary from 1700 cells/μl to 5000 cells/μl). Neutrophil percentage > 70% is highly suggestive of prosthetic joint infection (range 65–80%). Fourteen level 2 studies addressed the diagnostic efficacy of synovial fluid white blood cell count and differential [19, 21, 52, 58, 60–63, 71, 76, 77, 80, 93, 94]. Aspiration of synovial fluid for white blood cell count has advantages. It can be performed in the outpatient setting, and the results do not appear to be influenced by antimicrobial treatment [94].

**9. Bacterial culture from joint aspiration has high diagnostic accuracy in detecting prosthetic joint infection.**

Level of evidence: 2.

Given the limitations of the preoperative diagnostic work-up in identifying the presence or absence of infection, fluid and tissue cultures of the suspected infection site are often necessary. Twenty-three level 2 studies in our systematic review showed moderate to high sensitivity (43.5–100%) and high specificity (81.2–100%) for cultures [19, 57, 60, 65, 75, 78, 80, 92, 95–108]. The culture of multiple intraoperative tissue samples is important for the differentiation of contamination from infection. Accordingly, we recommend using five tissue samples for microbial culture for both aerobic and anaerobic culturing. Cultures can reliably confirm the etiology of joint infection in cases where two or more tissue samples show growth of the same micro-organism. The incubation time should be at least 7 days, although some studies even advise an incubation time of 2 weeks [65, 72]. Cultures may be false-negative due to prior use of an antimicrobial agent, low number of organisms, use of an inappropriate culture medium, infection by a fastidious organism, or prolonged transport time to the microbiology laboratory. Therefore, it is advisable to stop antibiotic treatment at least 2 weeks before sampling.

**10. Measurement of the synovial biomarkers alpha-defensin, leukocyte esterase, interleukin-6, and C-reactive protein is useful in the detection of prosthetic joint infection.**

Level of evidence: 2.

In the last decade, numerous studies have addressed the evaluation of new biomarkers in synovial fluid. These biomarkers show promising results. In our systematic review, alpha-defensin showed very high sensitivity and specificity (95.5–100% and 95–100%, respectively) in three level 2 studies and two level 3 studies [62, 68, 109–111]. Alpha-defensin is produced locally in the joint and does not appear to be influenced by antibiotic treatment before diagnostic evaluation. However, this test is expensive and not yet available in every hospital. Some low-grade bacteria such as *Cutibacterium acnes* (formerly *Propionibacterium acnes*) are less likely to be demonstrated with alpha-defensin measurement. Another synovial biomarker, leukocyte esterase, has also demonstrated diagnostic value, with sensitivity of 66–100% and specificity of 77–100% in five level 2 studies [112–116]. Leukocyte esterase is tested on a colorimetric reagent pad. It has the advantages of simplicity and of providing real-time results. Nevertheless, the reagent strip cannot be adequately read in the presence of blood or other debris. IL-6 has shown better diagnostic performance in synovial fluid than in serum. Six level 2 studies demonstrated sensitivity of 62.5–97% and specificity of 85.7–100% [57, 66, 76, 111, 117, 118]. IL-6 promotes osteoclast activation, consequently leading to prosthetic loosening. The costs are fairly high, and this test is not available in every hospital. CRP can be measured in serum and in synovial fluid. Synovial CRP showed diagnostic value, with sensitivity of 70–97.3% and specificity of 78.6–100%, in eight level 2 studies [62, 77, 79, 81, 111, 118–120]. Routine laboratory equipment that assays serum CRP can be used for testing synovial CRP. Non-elevated CRP in synovial fluid does not rule out PJI, as CRP is produced in the liver and not in the joints. Care must be taken to take physical examination and history into account, because CRP is a nonspecific marker of acute inflammation.

**11. Biopsy of periprosthetic tissue for histology and cultures can be performed for preoperative diagnosis in cases where ESR and/or CRP are positive and aspiration is inconclusive or impossible to test (dry tap).**

Level of evidence: 2.

Biopsy is not advised in all cases, as it is an invasive diagnostic modality. Five papers showed high diagnostic value (sensitivity 79.1–100%, specificity 90–100%); therefore, biopsy can be used to rule in and rule out infection [66, 67, 81, 121, 122]. Knees are more easily biopsied than hips, as in the hip, only the neck and head of the prosthesis and the inlay of the acetabular cup are easily accessible. Moreover, biopsy is associated with risk of infection and vascular or nerve injury. Using the blind technique could cause damage such as scratches to the articulating prosthetic surface. Culturing of biopsied periprosthetic tissue can identify the causative microorganism, and as a result, the correct antimicrobial treatment and adequate bone cement for the arthroplasty can be chosen.

**12. Antibiotic therapy should be postponed or discontinued before pre- and intraoperative sampling.**

Level of evidence: 4.

In clinical practice, the dictum that antibiotics should be withheld before obtaining microbiological cultures is well recognised. Some studies have confirmed the impact of previous antibiotic therapy on the probability of obtaining a microbiological diagnosis of PJI.

In a prospective trial in 79 patients with PJI, culture of samples obtained by sonication of the explanted prostheses were compared with conventional culture of periprosthetic tissue for the microbiological diagnosis of prosthetic joint infection. The sensitivity of the tissue cultures decreased (from 77% to 48% to 41%) as the antimicrobial-free interval before surgery decreased (from more than 14 days, to 4–14 days, and to 0–3 days, respectively) [20]. This was confirmed in another study, in which the sensitivity of periprosthetic tissue and synovial fluid culture increased from 54% to 73% when the patients who had received antimicrobial agents were excluded [93].

Another study found a statistically significant difference in the rate of positivity of biopsy specimen bacterial cultures between patients exposed and not exposed to previous antibiotic therapy [123].

Similarly, some studies reported higher sensitivity of synovial fluid culture in patients who did not take antibiotics before the procedure as compared with patients exposed to antibiotics [93, 124].

**13. Antibiotic therapy should not be discontinued before white blood cell scintigraphy.**

Level of evidence: 4.

The issue of whether on-going antibiotic treatment may interfere with the diagnostic accuracy of WBC scintigraphy has been widely disputed, yet there are no clinical comparative studies in patients with and without antibiotic treatment. Therefore, this information can only be extrapolated indirectly from studies that have included patients at diagnosis treated with antibiotics or from studies in patients during follow-up of therapy. Indeed, there are a number of studies of this kind in osteomyelitis (including peripheral bone osteomyelitis, prosthetic joint infection, and diabetic foot osteomyelitis) [125–131], soft tissue infections [132–136], inflammatory bowel diseases [137, 138], and cardiovascular infection [139, 140]. If taken together, approximately half of them conclude that antibiotic treatment reduces diagnostic accuracy [131, 135, 136, 138], and the other half report that it does not [125–130, 132, 137, 139]. This is based on the pooled sensitivity and specificity that seems to be reduced or unaffected with respect to patients at diagnosis without antibiotic therapy. Nevertheless, if we look only at only those papers in which the final diagnosis was made by histological evaluation of bone biopsy, we may conclude that WBC scintigraphy has the same diagnostic accuracy either before or during antibiotic treatment. This is particularly convincing for the diabetic foot infection [129, 130] and less convincing for prosthetic joint infections, mainly because of the results obtained by Chik et al. [131].

In summary, there is not sufficient evidence in the literature to reach a definitive conclusion on the impact of antibiotic treatment on the accuracy of WBC scintigraphy in PJI.

**14. Computed tomography can be effectively used to diagnose PJI.**

Level of evidence: 2.

Computed tomography has traditionally been used to evaluate orthopaedic implants. Striking artefacts caused by the interaction of the x-ray beam with the metallic hardware are now markedly reduced thanks to recent technical improvements. On CT, joint distension, fluid-filled bursae, and soft tissue collections are the main findings. Cyteval et al. reported that CT demonstrated 100% sensitivity, 87% specificity, and 89% accuracy when at least one soft tissue abnormality was used as an infection criterion, and 83% sensitivity, 96% specificity, and 94% accuracy when joint distention was used as infection criterion [141]. Absence of joint distension had 96% negative predictive value. Fluid collection in muscles and peri-muscular fat demonstrated 100% positive predictive value. Periosteal new bone has shown high specificity (100%) but low sensitivity (16%) for infection. Bone lucency around the implant is not a criterion for diagnosing infection, although in infected patients, CT may demonstrate a “more aggressive, ill-defined” lucency [142, 143]. Detection of a sinus tract may be difficult on CT.

**15. The diagnostic accuracy for three-phase bone scintigraphy in patients with suspected infection within the first 2 years after hip or knee prosthesis placement is low.**

Level of evidence: 2.

Three-phase bone scintigraphy is the most widely used screening modality for the diagnosis of PJI. However, it is common knowledge that the technique is highly sensitive but has low specificity for PJI, since any cause of increased bone formation (e.g. physiological bone remodelling or aseptic prosthetic loosening) shows increased periprosthetic activity on the bone scan. This physiological bone remodelling probably takes place in the first years after joint replacement surgery and is also dependent on the type of prosthesis and whether the prosthesis was cemented or uncemented.

The results of our search yielded 89 papers, of which 14 were included. After reading these papers thoroughly, we included only five papers that investigated the role of three phases in bone scan after joint replacement [144–148], four of which were reviews/overviews/expert opinions, and only one paper [144] reported the exact time between first surgery and bone scan (median duration 21 months in 39 patients after a unicompartmental knee replacement [UKR]). The authors found sensitivity of 50% and specificity of 71% for infection and concluded that there is no evidence to support the routine use of bone scan in clinical decision-making for patients with a painful UKR.

Experts agree that the diagnostic accuracy for three-phase bone scan in patients with suspected infections within the 5 years after hip or knee prosthesis placement is low. Van der Bruggen et al. [43] mention that in post-traumatic patients and after surgery, specificity is extremely low, around 35%. During this period, it is better to immediately perform another imaging technique, preferably white blood cell scintigraphy.

**16. In the case of negative three-phase bone scintigraphy, a diagnosis of prosthetic joint infection can be excluded.**

Level of evidence: 2.

Bone scintigraphy is widely used as first imaging modality when there is suspicion of a low-grade PJI. All three phases (perfusion, blood pool, and late phase depicting the osteoblastic activity) are necessary when an infection is suspected. Although a positive bone scan can be the result of any cause of increased osteoblast activity, a negative bone scan in all three phases means that there is no increased perfusion and no increased osteoblastic activity. It has been stated, therefore, that a negative three-phase bone scan rules out a diagnosis of PJI.

We conducted a literature search and 76 papers were retrieved, of which 15 were eventually included for thorough reading.

Only four research studies focused on the negative predictive value of the three-phase bone scan. Ikeuchi et al. [149] investigated the role of the bone scan in 15 patients after joint arthroplasty. They concluded that when the three-phase bone scan is negative, residual infection around the cement spacer is unlikely. Nagoya et al. [150] concluded that a negative three-phase bone scan suggests a low probability of periprosthetic infection and that one-stage revision could be considered in these cases. Volpe et al. [151] showed that a diagnostic algorithm using bone scan as a first screening modality had high diagnostic accuracy. Unfortunately, only seven patients had a negative bone scan, and timing between primary surgery and scans was not mentioned. Trevail et al. [152] investigated an imaging algorithm in 235 consecutive patients, of which only 17 were ultimately diagnosed with a proven infection. The authors also initially performed a bone scan. If the bone scan was negative, then infection was excluded and no WBC scan was performed. Using this algorithm, they found sensitivity of 80% and specificity of 99.5%. They did not mention, however, how many patients had a negative bone scan and in how many patients a WBC scan was performed.

Other included papers (overviews, reviews, and expert opinion) are in agreement that a study with normal findings (no increased perfusion or blood pool, no periprosthetic uptake in the late phase) can be considered strong evidence against the presence of an infection [146, 153–156].

**17. In the case of a positive three-phase bone scan, the addition of white blood cell scintigraphy leads to high diagnostic accuracy for PJI.**

Level of evidence: 2.

A positive three-phase bone scintigraphy can have several causes, including PJI, physiological bone remodelling, or aseptic loosening of the prosthesis. Therefore, other investigations must be performed to further differentiate between infection, reactive changes, and loosening. To determine whether WBC scintigraphy, when added to the diagnostic flowchart of a positive three-phase bone scintigraphy, leads to higher diagnostic accuracy, we conducted a PubMed search with the following terms: “(prosthetic OR prosthesis OR joint infection)) AND (bone scan OR MDP OR HDP OR bone scintigraphy)) AND (WBC scan OR WBC scintigraphy OR white blood cell scan OR white blood cell scintigraphy OR leukocyte scintigraphy)) AND diagnosis of infection”; 44 papers were retrieved, of which 19 were eventually included for thorough reading.

Only two research papers were found that specifically studied whether the addition of WBC scintigraphy after a positive bone scan leads to high diagnostic accuracy. Volpe et al. [149] showed that a diagnostic algorithm using the bone scan as first screening modality, and, when positive, the LeukoScan, achieved high diagnostic accuracy. Unfortunately, only seven patients had a negative bone scan, and timing between surgery and scans was not mentioned. Trevail et al. [152] also investigated an imaging algorithm in 235 consecutive patients, of which only 17 were ultimately diagnosed with a proven infection. The authors also performed an initial bone scan, which, if positive, was followed with a WBC scan. The use of this algorithm led to sensitivity of 80% and high specificity of 99.5%. Not mentioned, however, was how many patients had a negative bone scan and in how many patients a WBC scan was performed.

We also agree that another imaging modality is necessary in the case of a positive three-phase bone scan in patients with suspected PJI [3, 43, 157–160], and that WBC scintigraphy is the first nuclear imaging modality of choice in these cases because of its high diagnostic accuracy.

**18. In the case of negative white blood cell scintigraphy, the probability of prosthetic joint infection is low.**

Level of evidence: 2.

WBC scintigraphy has been described by many authors as highly sensitive and very specific for the diagnosis of PJI. However, there is still some concern whether low-grade chronic PJI with small amounts of bacteria on the biofilm can lead to false-negative WBC scan results.

We conducted a literature search to look at the diagnostic accuracy of WBC scintigraphy in patients with suspected PJI, and we retrieved 97 papers, of which 20 were eventually included for thorough reading and 12 after thorough reading.

The largest patient series included is from Kim et al. [161], who found NPV of 92% in 164 patients with suspected PJI (71 hip, 93 knee). Glaudemans et al. [154] used a time-decay dual-time-point imaging protocol and found NPV of 94% in 67 patients with suspected hip prosthesis infection, and NPV of 96% in 71 patients with suspected knee prosthesis infection. The same protocol was used by Erba et al. [162]. They report NPV of 93% in both hip and knee prostheses in 44 and 40 patients, respectively. Older studies used other protocols, including WBC labelled with ^111^In or anti-granulocyte imaging, or a combination of WBC/bone marrow (BM) scintigraphy.

Sousa et al. [163] report NPV NPV of 100% using sulesomab. Jung et al. [164] found NPV of 100%, but this was in only 11 patients and using a combined imaging protocol (WBC/BM). Simonsen et al. used both ^99m^Tc- and ^111^In-labelled WBC, and found 94% true-negative results [165]. Only Basu et al. found lower NPV (85% in patients with suspected hip prosthesis infection), but they used an incorrect acquisition protocol with only one imaging time point between 18 and 24 h after re-injection [166]. In conclusion, expert opinions and most research studies indicate high negative predictive value for WBC scintigraphy, which is even higher when performed with the correct acquisition protocol and interpretation criteria.

**19.** ^**18**^ **F-FDG-PET in patients with suspected prosthetic joint infection has high sensitivity but lower specificity than white blood cell scintigraphy or anti-granulocyte antibody scintigraphy.**

Level of evidence: 2.

A PubMed search based on the above-listed MeSH terms revealed 15 papers, of which five (three original research articles and two systematic reviews) were selected for thorough reading. In addition, four papers on the diagnostic performance of WBC scintigraphy in prosthetic joint infections were added.

There are few articles directly comparing the diagnostic performance of FDG-PET and WBC scintigraphy. The study by Love et al. [34] reported significantly higher accuracy for combined bone marrow and WBC scintigraphy than for FDG-PET, independent of the interpretation criteria used for FDG-PET. Vanquickenborne et al. [167] reported similar sensitivity between WBC scintigraphy and FDG-PET (88%), but higher specificity for WBC scan compared with FDG-PET (100% vs. 78%). The study by Pill et al. [168] reported sensitivity and specificity of 95% and 93%, respectively, for FDG-PET, while the use of combined ^99m^Tc-sulfur colloid- and ^111^In-labelled WBC demonstrated sensitivity of 50% and specificity of 95%.

Reinartz et al. [32], in a systematic review, reported higher sensitivity but lower specificity for FDG-PET compared with WBC scintigraphy. In addition, the accuracy for FDG-PET was found to be slightly higher in hip prosthesis than in knee prosthesis. In another review article, Gemmel et al. reported pooled sensitivity and specificity of 84% for FDG-PET in PJI, with higher accuracy for hip than knee prosthesis [31]. The joint EANM/SNMMI guidelines for the use of FDG in inflammation and infection (expert opinion) reported overall sensitivity of 95% for FDG-PET and specificity of 98% for knee and hip PJI [160].

In all mentioned systematic reviews, the range for both sensitivity (28–91%) and specificity (34–97%) in the individual studies was quite large. This is largely attributable to the differences in study design and interpretation criteria (visual interpretation using pattern recognition), emphasizing the need for standardized reconstruction and interpretation criteria.

Although there are not many published papers that directly compare FDG-PET and WBC scintigraphy in prosthetic joint infections, several different interpretation criteria for FDG-PET have been tested, and all papers confirm the lower specificity of FDG-PET compared with WBC scintigraphy. Large prospective studies comparing the diagnostic performance of WBC scintigraphy and FDG-PET for prosthetic joint infections are anticipated.

**20. Anti-granulocyte scintigraphy is a good alternative to white blood cell scintigraphy, with similar sensitivity and specificity** (mentions human anti-mouse antibodies [HAMA], differences between Fab and IgG, repeatability, which one to use in function of pretest probability, etc.).

Level of evidence: 2.

An extensive PICO was performed using a combination of different terms, but no papers were found that directly compared the diagnostic accuracy of labelled white blood cells with anti-granulocyte antibodies for patients with suspicion of prosthetic joint infections. A direct comparison between ^99m^Tc-besilesomab- and ^99m^Tc-labelled white blood cells in peripheral osteomyelitis, including patients with PJI, was published by Richter et al. [169], with comparable results for the two imaging approaches [163, 170].

Two meta-analyses on the diagnostic performance of anti-granulocyte antibodies in prosthetic joint infections were performed by Pakos et al. [171] and Xing et al. [172], and revealed overall sensitivity of 83% and specificity of 79–80%. These results are comparable to radiolabelled white blood cells, with slightly lower specificity for antibodies. Considering the results of these meta-analyses, the use of anti-granulocyte antibodies can be regarded as a valid alternative in patients with suspicion of prosthetic joint infections.

**21. Hybrid SPECT/CT imaging can improve localization of infection (and diagnostic accuracy).**

Level of evidence: 2.

Since the introduction of hybrid imaging technologies in clinical practice, the diagnostic accuracy of conventional nuclear exams has drastically improved due to the complementary molecular/functional and morphological information. Even though current recommendations for interpreting white blood cell scintigraphy are based solely on planar images at consecutive time points, adding SPECT/CT in the case of a positive result would theoretically further increase the diagnostic accuracy. It is well established for other pathologies that adding SPECT/CT to planar images results in higher diagnostic accuracy because of better resolution and morphological information.

A PubMed search yielded 50 papers, of which five papers (three original research articles [161, 173, 174] and two systematic reviews [31, 175]) were included. Two small studies [173, 174] showed increased specificity for SPECT/CT compared with SPECT alone and demonstrated contributory value in up to 38%. The largest study, by Kim et al., also demonstrated improved diagnostic accuracy with the addition of SPECT/CT [161].

Despite limited published data on white blood cell SPECT/CT in patients with suspicion of prosthetic joint infections, the incremental value of SPECT/CT over planar imaging is obvious. Adding SPECT/CT increases diagnostic accuracy by better distinguishing bone infection from soft tissue infections through improved assessment of the extent of the infection. Therefore, SPECT/CT should be recommended in the case of a positive planar white blood cell scan.

**22. Semiquantitative analysis of WBC accumulation over time in WBC scan increases diagnostic accuracy for PJI.**

Level of evidence: 3.

Based on the assumption that radiolabelled WBC, once injected into the patient, will migrate over time to an infected area and will progressively accumulate there due to the presence of chemotactic factors, nuclear medicine physicians have thought to quantify the presence of WBC in suspected lesions at 3–4 h after injection and again at 20–24 h. This quantitation is expressed as a ratio between radioactivity in the suspected region (target) and radioactivity in a background area. If this target-to-background (T/B) ratio increases over time (i.e. T/B_20h_ > T/B_4h_), there is active WBC accumulation, which is interpreted as an infection. By contrast, if the ratio decreases over time (i.e. T/B_20h_ < T/B_4h_), there is only transient WBC accumulation in the lesion, and it is interpreted as a sterile inflammatory process. Only two works have systematically investigated these assumptions, however, in retrospective studies, using the histological/microbiological information or long-term clinical follow-up of patients as gold standard [154, 176]. Both studies demonstrated a clear improvement in diagnostic accuracy for the semiquantitative method over simple qualitative image evaluation, particularly if the calculation of the T/B ratio was performed in both delayed images (3-4 h) and late images (20-24 h), and if the contralateral tissue was used as background instead of the ipsilateral bone marrow or ipsilateral iliac bone (often not available in the case of knee prosthesis). Glaudemans et al. showed that the T/B should show an increase of at least 10% to be considered a reliable indicator of granulocyte accumulation over time [154].

Other studies have applied a similar semiquantitative method, not only for prosthesis infection but also for soft tissue infection [161], post-traumatic osteomyelitis [176], and diabetic foot infection [35], or even with other radiopharmaceuticals such as FDG [34, 35, 177]. All studies were in agreement on the usefulness of semiquantitative evaluation of WBC accumulation in infected lesions over time. In particular, Van Acker et al. [178] emphasized that when only a qualitative approach is used, there is no benefit regardless of whether the analysis is done with or without a grading. It is worth mentioning that a study by Glaudemans et al. in a large number of patients demonstrated that semiquantitative analysis of the T/B ratio was particularly useful in those cases with a doubtful qualitative interpretation [154].

**23. Combining WBC scan with bone marrow scan increases diagnostic accuracy for PJI detection.**

Level of evidence: 2.

The use of combined WBC scintigraphy (using either ^99m^Tc-HMPAO-WBC or ^111^In-oxine-WBC) and bone marrow scintigraphy (with radiolabelled colloids) has been studied by many authors. This technique allows us to reduce the number of false-positive cases at WBC scintigraphy due to WBC accumulation in areas of expanded/displaced bone marrow by providing a map of bone marrow activity. It is particularly useful in cases of suspected PJI and in patients with a doubtful qualitative or semiquantitative analysis of WBC scintigraphy.

With few exceptions, the cumulative results obtained from papers describing this combined technique are remarkably consistent, with reported diagnostic accuracy ranging from 83% to 98% for both ^111^In-oxine-WBC and ^99m^Tc-HMPAO-WBC and for both hip and knee prosthesis infections [31, 34, 155, 165, 179–183]. Pill et al. in 2006 [168] and Joseph et al. in 2001 [184] reported low sensitivity (50% and 46%, respectively) but very high specificity (95% and 100%, respectively). The reason for the low sensitivity in these studies was that most of the examined infections were subacute or chronic, and/or microbiological data were often lacking. Given these few exceptions, it appears that combined in vitro labelled leucocyte/bone marrow scintigraphy (LS/BMS) has overall diagnostic accuracy of >90% and is the imaging modality of choice for diagnosing PJI.

Most importantly, the acquisition protocol and the interpretation criteria for WBC scintigraphy differed dramatically in the reported studies, thus emphasizing the importance of the combined LS/BMS technique.

Today, standardized acquisition and interpretation criteria are being disseminated and utilised across Europe, thus further reducing inter-observer variability and increasing the diagnostic accuracy and reproducibility of WBS scintigraphy. This will certainly help to reduce the number of doubtful cases in which to apply additional BMS scintigraphy.

**24. MRI is wholly feasible in patients with suspected PJI.**

Level of evidence: 2.

The presence of a joint prosthesis does not represent a contraindication to MRI. Traditionally, joint implants were considered potentially limiting to the outcome of an MR examination, due to a high quantity of susceptibility artefacts that could be generated by the metal itself. More recently, the advent of prostheses made with less ferromagnetic alloy materials and the technological advancements in MR sequences (metal artefact reduction sequences [MARS], slice encoding for metal artefact correction [SEMAC], and multi-acquisition with variable-resonance image combination [MAVRIC]) have made MRI wholly feasible in patients with joint implants, with artefacts largely confined to the area of the implant itself [185–187]. The diagnostic performance of MRI in PJI is addressed in statement 25.

**25. MRI demonstrates high diagnostic performance in detecting clinically suspected PJI, with no ionizing radiation.**

Level of evidence: 2.

Despite the widespread use of this protocol in clinical practice, most papers on MRI and joint prosthesis were focused on the technical feasibility of this examination. Only three papers specifically aimed to evaluate prosthesis infection. In the knee, Plodowski et al. found 86–92% sensitivity and 85–87% specificity for infection, while Li et al. found 65–78% sensitivity and 98–99% specificity. In the hip, He et al. found 94% sensitivity and 97% specificity. Like ultrasound, MRI is an imaging modality that does not use ionizing radiation. This fact should be given particular consideration, especially in these patients, who typically need repeated examinations [44–47].

## Conclusions and final recommendations

**Fig. 1:**
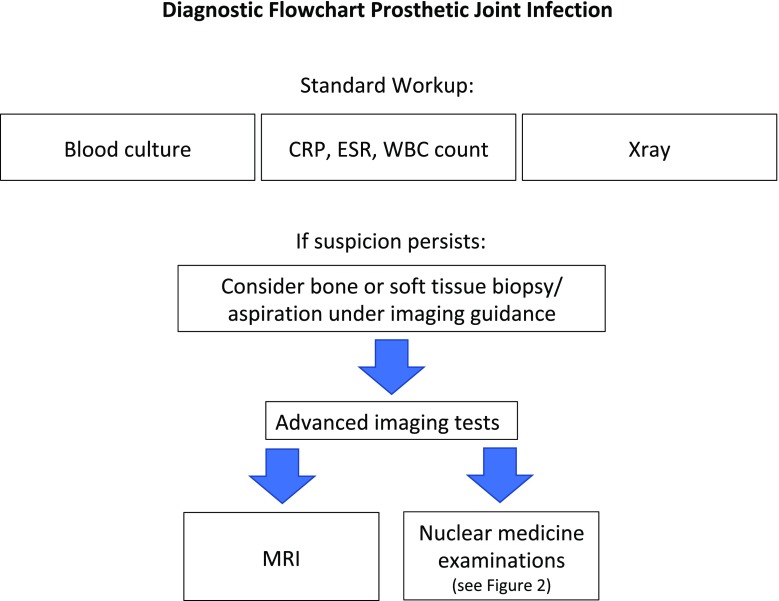
The suggested initial diagnostic steps to undertake in the case of suspicion of a PJI, based on published evidence. Some tests can be repeated (i.e. blood cultures, bone biopsies, or soft tissue biopsies). Serological tests (CRP, WBC count with differential, and ESR) should be performed over time, since the overall increasing or decreasing trend is more important than a single value. The choice of an advanced diagnostic test depends on availability, costs, radiation burden, and operator experience (see Tables 1 and 2). Synovial biomarkers and cultures can be better performed after sonication of tissue samples. They have high accuracy for infection but need to be integrated with advanced imaging modalities to study the bone and soft tissue status and the extent and severity of the infection

**Fig. 2:**
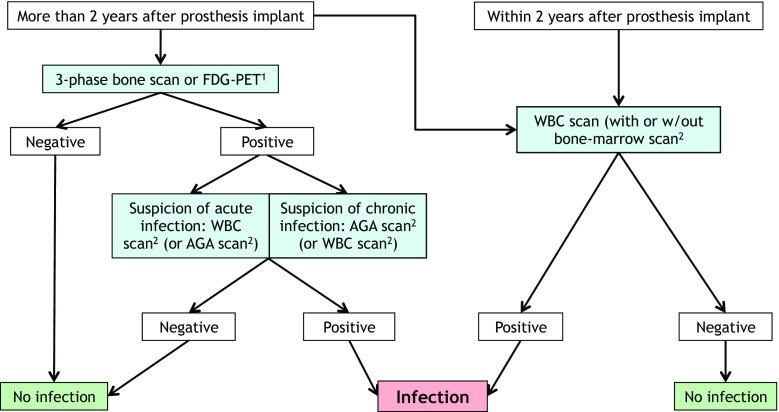
The suggested path to undertake when nuclear medicine procedures are considered for suspicion of a PJI, based on published evidence and expert opinion. Initial stratification is based on time after implant (more or less than 2 years). This is particularly true for hip and shoulder prostheses, but knee prosthesis may require up to 5 years post-implant to reduce physiological inflammation. Some differences may also depend on the type of prosthesis (cemented or not), with cemented prostheses having a shorter post-implant time for physiological inflammatory reaction. ^1^FDG-PET has higher sensitivity than specificity, mainly because of false-positive inflammatory uptake in the case of aseptic loosening and/or recent surgery. This is why it is better to exclude an infective process in chronic painful joints. Nevertheless, despite the lack of standardized image interpretation criteria available, FDG-PET has also been proposed in early acute phases of infections. ^2^WBC scan has higher sensitivity and specificity than AGA scan, FDG-PET, and MRI, and is preferred when available and indicated for the patient. The methodology for these nuclear medicine scans is extremely important (usually by acquiring three sets of images corrected for isotope decay), and we refer to the procedural guidelines published by the EANM Committee on Infection/Inflammation. It can be combined with bone marrow scintigraphy to further increase specificity

The proposed flowchart is shown in Fig. 1. Regarding the advanced diagnostic tests, there are too few well-designed papers (with a high level of evidence) that directly compare radiological and nuclear medicine techniques. When choosing an advanced diagnostic test, further stratification should be performed based on the pretest probability of infection and local availability and experience. Overall, considering the diagnostic performance of all modalities, both WBC scan (with or without bone marrow scan) or anti-granulocyte scintigraphy (preferably with IgG) and non-contrast MRI can be performed as an initial investigation (Fig. 2).

## Evidence-based diagnostic flow charts

Based on the above-mentioned statements and evidence from the published literature, we have developed a diagnostic flow chart, shown in Fig. 1, correlated with Tables 1 and 2.

**Table 1:** Advanced radiological techniques

	Ultrasound	Computed tomography	Magnetic resonance
Pros	May be useful in monitoring soft tissue extension of infection and for soft tissue biopsies Widely available and low cost	Needed as a guide for bone biopsy Widely available and medium cost	High diagnostic accuracy using new sequences without interference from the prosthesis Widely available and medium cost Radiation-free
Cons	Low sensitivity and specificity for bone infection	Possible striking artefacts due to the metal nature of prosthesis Overall lower diagnostic accuracy than MR High radiation exposure Possible side effects from contrast agent	Peri-implant edema may occasionally suggest false-positive findings

**Table 2:** Advanced nuclear medicine techniques

	^99m^Tc-MDP/HDP bone scan	^99m^Tc-anti-granulocyte scan (IgG/Fab AGA)	^99m^Tc-HMPAO/^111^In-oxine-WBC scan	[^18^F]FDG-PET/CT
Pros	High sensitivity Useful as screening method in chronic infections Widely available and low cost	High sensitivity and specificity; however, generally lower than for WBC scan Data support the preferential use of IgG over Fab in chronic infections. Widely available and medium cost Often to be used coupled with bone marrow scan and/or bone scan	High sensitivity and specificity Data support preferential use in acute infections Poor availability and medium cost Often to be used coupled with bone marrow scan SPECT/CT images improve accuracy	High sensitivity
Cons	Low specificity Moderate radiation exposure	Possible contraindications for IgG and HAMA induction Moderate radiation exposure IgG scan requires a late acquisition at 20 h p.i.	Moderate radiation exposure Always requires a late acquisition at 20 h p.i. Blood manipulation Needs an approved laboratory and method and trained personnel	Low specificity High radiation exposure Difficult interpretation of images Poor availability and high cost

In some cases, the flow was integrated by consensus opinion amongst the experts, since not all steps are always clearly deducible from the literature or from level 1–2 articles. The flow chart does not take into consideration socioeconomic factors or the availability of diagnostic methods. It also presumes that all exams are optimally performed (possibly following procedural guidelines published by each European or national society, when available) and by expert professionals.

## Electronic supplementary material

ESM 1 (DOCX 23 kb)

ESM 2 (DOCX 67 kb)

ESM 3 (DOCX 44 kb)

## Abbreviations

PJI: Prosthetic joint infection
CRP: C-reactive protein
ESR: Erythrocyte sedimentation rate
WBC: White blood cells
NSAIDs: Nonsteroidal anti-inflammatory drugs
PICO: Population/problem – Intervention/indicator – Comparator – Outcome
CT: Computed tomography
EANM: European Association of Nuclear Medicine
ESR: European Society of Radiology
EBJIS: European Bone and Joint Infection Society
ESCMID: European Society of Clinical Microbiology and Infectious Diseases
OCEBM: Oxford Centre for Evidence-based Medicine
MRI: Magnetic resonance imaging
FDG: Fluor-deoxy-glucose
Tc: Technetium
PET: Positron emission tomography
AGA: Anti-granulocyte antibodies
mAbs: Monoclonal antibodies
SPECT: Single-photon emission computed tomography
US: Ultrasound
HDP: Hydroxymethylene diphosphonate
MDP: Methylene diphosphonate
HMPAO: Hexamethylpropyleneamine oxime
T/B: Target-to-background ratio
Oxine: 8-hydroxyquinoline
MARS: Metal artefact reduction sequences
MAVRIC: Multi-acquisition variable-resonance image combination
SEMAC: Slice encoding for metal artefact correction
