# Supplementary material for: Consensus document for the diagnosis of prosthetic joint infections: a joint paper by the EANM, EBJIS, and ESR (with ESCMID endorsement)
Source: Eur J Nucl Med Mol Imaging. 2019 Jan 26;46(4):971–88. doi: 10.1007/s00259-019-4263-9 (PMC6450843; doi:10.1007/s00259-019-4263-9)
Supplement: Supplementary file 1 — (DOCX 23 kb) [file 259_2019_4263_MOESM1_ESM.docx]

**Appendix 1. Diagnosis of PJI**

***Clinical assessment, symptoms and signs of PJI***

Clinical signs of early infections are persisting local pain, erythema, oedema, wound healing disturbance e.g.leakage, large haematoma and fever. Persisting or increasing joint pain and early loosening are also important symptoms and signs, particularly for delayed infection. Therefore, such infections are often difficult to distinguish from aseptic failure. Late infections present either with a sudden onset of systemic symptoms (in about 30%) or as a subacute infection following unrecognised bacteraemia (in about 70%)[6]. The most frequent primary (distant) focus of implant-associated infections is the skin, followed by the respiratory tract, dental and urinary tract.

***Value of laboratory parameters***

Laboratory parameters of inflammation (blood leukocyte count, erythrocyte sedimentation rate, serum C-reactive protein (CRP) and procalcitonin) are often modified during bone and prosthetic joint infection as well as in aseptic loosening, and therefore they are not sufficiently discriminative to predict the presence or absence of infection. Serum CRP, moreover, can frequently increase after implant of a prosthetic joint; for this reason, there is a need for repetitive CRP assays and to follow its trend over time. A sharp CRP increase after surgery or during follow-up is highly suggestive of the presence of infection but not sufficient in itself to evaluate the severity and extent of the infection, for which there is a need for additional approaches, including imaging. A patient with fever and/or persistently elevated CRP and ESR or WBC count is considered to have a high pre-test probability of infection thus influencing the choice of the imaging modality to be performed at first.

***Microbiological diagnosis***

It is recommended to perform a blood culture for both aerobic and anaerobic germs in febrile patients in addition to a joint aspiration, when possible.

Obtaining a microbiological diagnosis of PJI is extremely important for choosing the appropriate antimicrobial treatment. The treatment of these infections should be guided by microbiological findings to improve the outcome of patients. The most reliable tool for identifying causative bacteria is represented by cultures from periprosthetic tissue. Cultures from aspirated synovial fluid may also be performed. It has been reported that pathogens of PJI may be detected in synovial fluid in 45–100% of cases [17] and in peri-proshetic tissue samples in 65-94% [18, 19].

Cultures from superficial wounds or sinus tracts are not reliable in identifying the causative bacteria since these cultures may represent microbial skin colonization instead of implant-associated infection.

Cultures from intra-operative tissue may give a false negative result for several reasons: use of inappropriate mediums, inadequate incubation time, or loss of viability during transport of the specimen, adherence of bacteria to the prosthesis itself within the surrounding glycocalyx; prior antimicrobial exposure.

The use of sonication technique (sample biofilm bacteria on the surface of removed hip and knee implants placed in solid containers) may improve the sensitivity of cultures. A prospective trial compared culture of samples obtained by sonication of explanted hip and knee prostheses to dislodge adherent bacteria from the prosthesis with conventional culture of periprosthetic tissue for the microbiologic diagnosis of PJI among patients undergoing hip or knee revision or resection arthroplasty. The sensitivities of periprosthetic-tissue and sonicate-fluid cultures significantly differed (60.8% and 78.5%, respectively) the specificities were of similar value (99.2% and 98.8%, respectively). Fourteen cases of PJIs were detected by sonicate-fluid culture but not by prosthetic-tissue culture. The sensitivities of peri-prosthetic tissue and sonicate-fluid culture significantly differed also in patients receiving antimicrobial therapy within 14 days before surgery (45% and 75%, respectively) [20].

***Joint aspiration and its limitations***

Detection of causative microorganisms can be achieved by pre-operative aspiration of fluids and intra-operative culture of at least three tissue samples. Swab samples are not sensitive enough to be performed. Antimicrobial treatment has been suggested to be stopped at least two weeks before sampling for microbiological culture. In case of explanted devices, sonication can increase the sensitivity of the culture, by dislodging microorganisms from the surface of the device. This effect is more evident in patients who received prior antimicrobial treatment. Molecular methods can further improve the microbiological diagnosis [21].

***Radiologic imaging methods and its limitations***

Conventional radiographs are normally used to evaluate joint prostheses after implantation and follow-up to have a general overview of the implant and to detect the presence of other causes of pain. However, diagnostic performance of conventional radiographs in detecting PJI is low. Further, conventional radiographs frequently become positive when more than 30-50% of bone mass has been lost and abnormalities around the implant are usually non-specific for infection. In addition, about 50% of conventional radiographs remain normal also in presence of an infection. Regarding ultrasound, controversial results have been reported in the detection of PJI, however it can be used as a guide for aspiration procedures. US can be effectively used to detect the presence of peri-prosthetic fluid collections, being also occasionally able to differentiate abscesses from aseptic collections [22]. Also, US is able to track the presence of sinus tracts within soft tissues. The main advantages of US are its wide availability, low cost and the possibility of being performed at bedside. It can also be repeated when needed, as it does not use ionizing radiation [22]. Computed tomography (CT) has been reported to have good diagnostic performance in the detection of PJI, with up to 84% accuracy; however, the use of ionizing radiations should limit its use. CT is also the imaging modality of choice as a guide to perform bone biopsies. Although most papers have been focused on technical feasibility, magnetic resonance imaging (MRI) has been reported highly sensitive (92%) and specific (99%) to diagnose PJI, with the great advantage of not using ionizing radiations or contrast agents [23-25].

***Nuclear medicine imaging techniques and limitations***

Nuclear Medicine planar imaging is not affected by metallic hardware, and can play an important role in the diagnosis of prosthetic joint infection. The evaluation of hybrid images (SPECT/CT) can also be performed after imaging reconstruction without attenuation correction, with an overall accuracy ranging from 88 to 98% (being highest for WBC combined with bone marrow imaging).

The use of anti-granulocyte mAb scintigraphy has also been successfully used in PJI. Nevertheless, the image acquisition protocols are not always identical and in addition, there may be some variation in the criteria used for image interpretation.

*Bone scintigraphy*

It is performed by injecting a derivative of a diphosphonate radiolabelled with ^99m^Tc; although nowadays also [^18^F] fuoride is available for PET studies but its poor availability and high cost are important limiting factors.

After a prosthetic implant the bone is obviously damaged and some remodelling will occur over the following months. Cemented prosthesis may induce less bone stimulation then bio-inductive prosthesis, although it has been suggested by many authors to avoid the use of bone scintigraphy for the diagnosis of infection within 2 years after hip prosthesis implant and up to 5 years after knee prosthesis implant.

Bone scan has a very high sensitivity, although low specificity for infection, and can, therefore, be used to exclude the presence of infection when it is negative.

It is also useful to show bone abnormalities in case of mobilization of the prosthesis, particularly if hybrid SPECT/CT technique is used. Recently, the EANM Bone & Joint Committee has published procedural guidelines on how to perform this modality at best for each pathology [26].

*White Blood Cell scintigraphy*

Taking into account the different biodistribution of labelled WBC in blood, bone-marrow, infection and sterile inflammation, 3 sets of images must be generally acquired: “early images” (within 30 minutes and 1 h p.i.), “delayed images” (between 2 h and 4 h p.i.) and “late images” (between 20 h and 24 h p.i.).According to the clinical indication, whole-body, planar and, if appropriate, SPECT (or SPECT/CT) images should also be performed at 3-4 h and 20-24 h.

The complete study requires several scans over 2 days and, unfortunately, it is not easily available in every nuclear medicine center. WBC labelling is regulated by several national rules and guidelines [27, 28], with minor variations between countries. The overall diagnostic accuracy of this technique is above 95% for PJI and the exam constitutes the gold standard diagnostic technique for PJI.

*Anti-granulocyte antibody scintigraphy*

With mAbs, image protocols differ between complete and fragmented antibodies [29]: images with complete ^99m^Tc-anti-NCA-95 antibody (besilesomab) should be performed at 2-4 h p.i. and 16-24 h p.i. in planar whole body technique because a significant increase in sensitivity and specificity will be achieved with delayed 24 h images due to higher target to background ratios (T/B). Planar images can be performed with the same acquisition protocol as WBC. The best time point for SPECT images is 4-6 h after injection but another SPECT at 16-24 h p.i can also be performed if required, similarly to WBC scan.

With ^99m^Tc-anti-NCA-90 (Fab') antibodies (sulesomab), images should be performed 1 h p.i. and 4-6 h p.i. also with planar whole body images, better if supported by three-phase a bone scan or a boone-marrow scan. SPECT of suspected central bone infection should be performed at 4-6 h p.i.

*Bone marrow scintigraphy*

Bone marrow scintigraphy is usually performed when doubtful WBC scans for bone and prosthetic joint infections are obtained. These are performed at the end of the WBC/mAb study (same day of the 24 h image or in the next few days) by injecting intravenously (i.v.) about 185 MBq (5 mCi) of ^99m^Tc- colloids (colloids of greater than 500 nm are recommended) and acquiring images of the region of interest after a minimum of 20-30 minutes and a maximum time of 6 hours p.i.

*Fluor-deoxy-glucose PET*

Considering the available published data, it is unclear if [^18^F]FDG imaging offers any significant advantage over radiolabeled WBC or anti-granulocyte monoclonal antibodies in the study of joint prosthetic infections [30, 31]. Some interpreting criteria have been proposed by Reinartz et al. [32], Chacko et al. [33], Love et al. [34], Familiari et al. [35] and Stumpe et al. [36] for painful hip arthroplasties but the overall accuracy is below 90% and has not been confirmed by others [37]. For sure, visual interpretation using these criteria may be more reliable than quantitative (SUV) analysis, which is not recommended.

# ***Hybrid imaging techniques***

The advent of hybrid imaging technologies combining molecular/functional and anatomical information has significantly increased the diagnostic accuracy of conventional nuclear exams by increasing sensitivity and specificity and reducing the number of equivocal lesions. This hybrid technology has redefined the work-up of our patients and has influenced patient management. The hybrid imaging techniques have in general a better spatial resolution compared to conventional planar images and allow a more accurate localization and assessment of disease extent.

SPECT/CT could be an integral part of a conventional WBC/mAbs scintigraphy mainly aiming to better distinguish bone from soft tissue infections and to more accurately assess the extent of the infectious process [38-41][^18^F]FDG-PET/CT can be considered as a first-line diagnostic tool for evaluating a variety of inflammatory and infectious diseases [42, 43], however, its role in orthopedic infections has not been clearly established especially not in the post-operative setting or in the presence of prosthetic joints or osteosynthesis.

The latter can be attributed to metal artefacts that negatively influence the exact localization of the infection as well as the low specificity of FDG that makes it difficult to distinguish a sterile inflammation from inflammation caused by infection.

More recently, the introduction of PET/MRI has emerged as a powerful diagnostic tool, but so far no reports have been published on its value in PJI. The general advantages of MRI compared to CT include a better evaluation of soft tissue and the lack of radiation burden, and MRI sequences that avoid artefacts of metallic implants are now widely available [23-25, 44-47].

Finally, it is worthwhile mentioning that one should always keep in mind that the final decision for a particular imaging technique will also be highly dependent on the local availability, time, costs and expertise.
