# Supplementary material for: Consensus document for the diagnosis of prosthetic joint infections: a joint paper by the EANM, EBJIS, and ESR (with ESCMID endorsement)
Source: Eur J Nucl Med Mol Imaging. 2019 Jan 26;46(4):971–88. doi: 10.1007/s00259-019-4263-9 (PMC6450843; doi:10.1007/s00259-019-4263-9)
Supplement: Supplementary file 2 — (DOCX 67 kb) [file 259_2019_4263_MOESM2_ESM.docx]

**Appendix 2. Concerns on the use of ionizing radiations**

Ionizing radiations are considered a potential risk for patients undergoing radiological and nuclear medicine examinations. In 2014, the EuroSafe Imaging Campaign was launched by the ESR to promote and strengthen medical radiation protection across Europe [http://www.eurosafeimaging.org/]. Similar concern has always been taken into consideration by the EANM with a significant reduction of administered doses to patients, over the years, also thanks to the advances in technology and availability of ultra-sensitive scanners.

On the basis of previous directives, the European Union issued a document on safety standards for protection against the potential dangers deriving from medical exposure to ionising radiation (2013/59/EURATOM directive), [https://ec.europa.eu/energy/sites/ener/files/documents/CELEX-32013L0059-EN-TXT.pdf]. The principle of justification to medical exposure is clearly relevant and the physician must always takeinto account efficacy, benefits and risks as well as the possibility to obtain similar results by using alternative techniques not involving the use of ionizing radiations.
