# Supplementary material for: Consensus document for the diagnosis of prosthetic joint infections: a joint paper by the EANM, EBJIS, and ESR (with ESCMID endorsement)
Source: Eur J Nucl Med Mol Imaging. 2019 Jan 26;46(4):971–88. doi: 10.1007/s00259-019-4263-9 (PMC6450843; doi:10.1007/s00259-019-4263-9)
Supplement: Supplementary file 3 — (DOCX 44 kb) [file 259_2019_4263_MOESM3_ESM.docx]

# **APPENDIX 3. PICOs belonging to statements**

**PJI should be suspected when one or more of the following symptoms and signs are present: otherwise unexplained pain and/or fever, redness, swelling, scar inflammation, and movement limitations. These symptoms are (especially in the chronic phase) not specific and require other investigations.**

Level of evidence: 4

P: infection AND (joint OR prosthetic OR arthroplast*)

I: symptoms AND (pain OR fever OR redness OR swelling OR scar inflammation OR movement limitations)

C: -

O: diagnostic accuracy OR sensitivity OR specificity

(Prosthesis-Related Infections/diagnosis"[MAJR] AND "joints"[MeSH Terms] AND "infection"[MeSH Terms] AND "humans"[MeSH Terms] AND English[lang] AND "adult"[MeSH Terms])

All three together: 133

Included papers for thorough reading: 41

Included papers after thorough reading: 6

**2.**

**Sinus tract and purulent discharge are clear signs of Prosthetic joint infection.**

Level of evidence 5

P: infection AND (joint OR prosthetic OR arthroplast*)

I: symptoms AND (sinus tract OR purulent OR discharge OR fistula)

C: -

O: diagnostic accuracy OR sensitivity OR specificity

(Prosthesis-Related Infections/diagnosis"[MAJR] AND "joints"[MeSH Terms] AND "infection"[MeSH Terms] AND "humans"[MeSH Terms] AND English[lang] AND "adult"[MeSH Terms])

No papers published.

**3.**

**CRP and ESR should always be performed in patients suspected of prosthetic joint infection. A normal value does not rule out PJI.**

Level of evidence: 2

P: infection AND (joint OR prosthetic OR arthroplast*)

I: C-reactive protein OR CRP OR erythrocyte sedimentation rate OR ESR (title/abstract)

C: -

O: diagnostic accuracy OR sensitivity OR specificity

((ESR[Title/Abstract] OR CRP[Title/Abstract] OR C-reactive protein[Title/Abstract] OR erythrocyte sedimentation rate[Title/Abstract]) AND (infection) AND (arthroplast* OR prosthetic* OR joint) AND (sensitivity OR specificity OR diagnostic accuracy))

All three together: 233 articles

Included papers for thorough reading: 48 articles

Included papers after thorough reading: 33 articles

4.

**In case of fever, blood cultures should always be performed in patients suspected to have prosthetic joint infection for identifying causative bacteria**

Level of evidence: 5

P: Prosthesis-Related Infections/diagnosis AND joints

I: Infection and humans

C: -

O: -

((Prosthesis-Related Infections/diagnosis"[MAJR]) AND ("joints"[MeSH Terms]) AND ("infection"[MeSH Terms]) AND ("humans"[MeSH Terms]) AND (English[lang]) AND ("adult"[MeSH Terms]))

All three together: 133

Included papers for thorough reading: 41

Included papers after thorough reading: 5

**5.**

**Conventional radiographs are the first imaging modality to perform in patients with joint pain suspected of PJI for diagnosis and follow-up.**

Level of evidence: 2

P: hip OR knee OR joint AND implant OR prosthesis OR arthroplasty AND infection

I: X ray OR plain film OR conventional radiography

C: -

O: diagnostic performance OR diagnosis OR accuracy

(((hip OR knee OR joint) AND (implant OR prosthesis OR arthroplasty) AND (infection)) AND (xray OR plain film OR conventional radiography) AND (diagnostic performance OR diagnosis OR accuracy))

Papers found: 551

Included papers for thorough reading: 37

Papers selected: 3

**6.**

**Ultrasound can detect complications around the prosthesis, but capability of detecting infection is controversial.**

Level of evidence: 2

P: hip OR knee OR joint AND implant OR prosthesis OR arthroplasty AND infection

I: ultrasound OR ultrasonography

C: -

O: diagnostic performance OR diagnosis OR accuracy

(((hip OR knee OR joint) AND (implant OR prosthesis OR arthroplasty) AND (infection)) AND (ultrasound OR ultrasonography) AND (diagnostic performance OR diagnosis OR accuracy))

Papers found: 86

Included papers for thorough reading: 6

Papers selected: 2

**7.**

**Imaging guidance should be always used to perform joint aspiration or periprosthetic tissue biopsy.**

Evidence level: 2

P: hip OR knee OR joint AND implant OR prosthesis OR arthroplasty AND infection

I: aspiration

C: -

O: diagnostic performance OR diagnosis OR accuracy

(((hip OR knee OR joint) AND (implant OR prosthesis OR arthroplasty) AND (infection)) AND (ultrasound OR ultrasonography) AND (diagnostic performance OR diagnosis OR accuracy))

Papers found: 286

Included papers for thorough reading: 8

Papers selected: 3

**8.**

**Leukocyte counts and differential of synovial fluid have high diagnostic accuracy to detect PJI.**

Level of evidence: 2

P: infection AND (joint OR prosthetic OR arthroplast*)

I: leukocyte count OR white blood cell count OR differential AND synov*

C: -

O: diagnostic accuracy OR diagnosis infection

(((leukocyte count OR white blood cell count OR differential) AND (synov*)) AND ((infection) AND (arthroplast* OR prosthetic OR joint)) AND (sensitivity OR specificity OR diagnostic accuracy))

All three together: 112 articles

Included papers for thorough reading: 22 articles

Included papers after thorough reading: 14 articles

**9.**

**Bacterial cultures have high diagnostic accuracy to detect prosthetic joint infection.**

Level of evidence: 2

P: infection AND (joint OR prosthetic OR arthroplast*)

I: aspiration OR culture (title/abstract)

C: -

O: diagnostic accuracy OR sensitivity OR specificity

((Aspiration[Title/Abstract] OR culture[Title/Abstract]) AND (diagnostic accuracy OR sensitivity OR specificity) AND (infection) AND (joint OR prosthetic OR arthroplast*))

All three together: 539 articles

Included papers for thorough reading: 34 articles

Included papers after thorough reading: 23 articles

**10.**

**Measurement of the synovial biomarkers alpha-defensin, leukocyte esterase, interleukin-6 and C-reactive protein can be useful in the detection of prosthetic joint infection.**

Level of evidence: 2

P: infection AND (joint OR prosthetic OR arthroplast*)

I: (synov* OR biomarker) AND (alpha-defensin OR leukocyte esterase OR leucocyte esterase OR interleukin-6 OR C-reactive protein OR CRP)

C: -

O: diagnostic accuracy OR sensitivity OR specificity

((alpha-defensin OR leukocyte esterase OR leucocyte esterase OR interleukin-6 OR C-reactive protein OR CRP) AND (synov* or biomarker*) AND (sensitivity OR specificity OR diagnostic accuracy) AND (infection) AND (arthroplast* OR prosthetic OR joint))

All three together: 135 articles

Included papers for thorough reading: 45 articles

Included papers after thorough reading: 31 articles

**11.**

**Biopsy of peri-prosthetic tissue for histology and cultures can be performed for pre-operative diagnostics incase ESR and/or CRP are positive and aspiration is inconclusive or impossible to test (dry tap).**

Level of evidence: 2

P: infection AND (joint OR prosthetic OR arthroplast*)

I: biopsy OR tissue (title/abstract)

C: -

O: diagnostic accuracy OR sensitivity OR specificity

((biopsy[Title/Abstract]) AND (tissue[Title/Abstract]) AND (infection) AND (joint OR prosthetic OR arthroplast*) AND (diagnostic accuracy OR sensitivity OR specificity))

All three together: 33 articles

Included papers for thorough reading: 9 articles

Included papers after thorough reading: 5 articles

**12.**

**Antibiotic therapy should be postponed or discontinued before pre- and intra-operative sampling**

Level of evidence: 4

P: Prosthesis-Related Infections/diagnosis AND joints

I: infection AND humans

C: -

O: -

((Prosthesis-Related Infections/diagnosis"[MAJR] AND "joints"[MeSH Terms]) AND ("infection"[MeSH Terms]) AND ("humans"[MeSH Terms]) AND (English[lang]) AND ("adult"[MeSH Terms]))

All three together: 133

Included papers for thorough reading: 41

Included papers after thorough reading: 4

**13.**

**Antibiotic therapy should not be discontinued before white blood cell scintigraphy.**

Level of evidence: 4

P= PJI or prosth* infection

I= wbcscintigraphy or WBC scan or white blood cell scintigraphy or 111In-WBC or 99mTc-WBC

C= antibiotic or therapy or treatment or antimicrobial

O= diagnosis or diagnostic accuracy or accuracy

((PJI or prosth* infection) AND (wbc scintigraphy or WBC scan or white blood cell scintigraphy or 111In or 99mTc) AND (antibiotic or therapy or treatment or antimicrobial) AND (diagnosis or diagnostic accuracy or accuracy))

All together: 26 papers

Included papers for thorough reading: 5

Included papers after thorough reading: 2

**14.**

**Computed tomography can be effectively used to diagnose PJI.**

Level of evidence: 2

P: hip OR knee OR joint AND implant OR prosthesis ORarthroplasty AND infection

I: computed tomography OR computed assisted tomography

C: -

O: diagnostic performance OR diagnosis OR accuracy

((hip OR knee OR joint) AND (implant OR prosthesis OR arthroplasty) AND (infection) AND (computed tomography OR computed assisted tomography) AND (diagnostic performance OR diagnosis OR accuracy))

Papers found: 293

Included papers for thorough reading: 18

Papers selected: 3

**15.**

**The diagnostic accuracy for 3 phase bone scintigraphy in patients with suspected infections within the first 2 years after hip or knee prosthesis placement is low.**

Level of evidence: 1

P: patients with hip prosthesis OR knee prosthesis

I: three phase bone scan – bone scintigraphy – bone scan - HDP – MDP – DPD

C: -

O: diagnosis infection – diagnostic accuracy

((patients with hip prosthesis OR knee prosthesis) AND (diagnosis infection OR diagnostic accuracy) AND (three phase bone scan OR bone scintigraphy OR bone scan OR HDP OR MDP OR DPD))

All three together: articles 89

Included papers for thorough reading: 14

Included papers after thorough reading: 5

**16.**

**In case of negative three phase bone scintigraphy, the diagnosis of prosthetic joint infection can be excluded.**

Level of evidence: 2

P: hip prosthesis OR knee prosthesis OR joint arthroplasty

I: bone scan OR bone scintigraphy OR HDP OR MDP OR DPD or three phase bone scan

C: -

O: specificity OR negative for infection OR excludes infection

((hip prosthesis OR knee prosthesis OR joint arthroplasty) AND (bone scan OR bone scintigraphy OR HDP OR MDP OR DPD or three phase bone scan) AND (specificity OR negative for infection OR excludes infection))

All three together: articles 76

Included papers for thorough reading: 15

Included papers after thorough reading: 4

**17.**

**In case of positive three phase bone scan, the addition of white blood cell scintigraphy leads to high diagnostic accuracy for PJI.**

Level of evidence: 2

P: prosthetic OR prosthesis OR joint infection OR arthroplasty

I: bone scan OR MDP or HDP OR bone scintigraphy

C: WBC scan OR WBC scintigraphy OR white blood cells scan OR white blood cell scintigraphy

O: diagnosis of infection OR diagnostic accuracy

((prosthetic OR prosthesis OR joint infection OR arthroplasty) AND (bone scan OR MDP or HDP OR bone scintigraphy) AND (WBC scan OR WBC scintigraphy OR white blood cells scan OR white blood cell scintigraphy) AND (diagnosis of infection OR diagnostic accuracy))

Total: 44

Included papers for thorough reading: 19

Included papers after thorough reading: 11

**18.**

**In case of negative white blood cell scintigraphy, the probability of prosthetic joint infection is low.**

Level of evidence: 2

P: prosthetic OR prosthesis OR joint infection OR arthroplasty

I: WBC scan OR WBC scintigraphy OR white blood cells scan OR white blood cell scintigraphy

C: -

O: diagnosis of infection OR diagnostic accuracy

((prosthetic OR prosthesis OR joint infection OR arthroplasty) AND (WBC scan OR WBC scintigraphy OR white blood cells scan OR white blood cell scintigraphy) AND (diagnosis of infection OR diagnostic accuracy))

All three together: 97

Included papers for thorough reading: 20

Included papers after thorough reading: 12

**19.**

**^18^F-FDG-PET in patients suspected of prosthetic joint infection has high sensitivity but lower specificity than white blood cell scintigraphy or anti-granulocyte antibodies scintigraphy.**

Level of evidence: 2

P: prosthetic or prosthesis or joint

I: FDG or FDG-PET or PET-CT or fluorodeoxyglucose or positron emission tomography

C: wbc or scintigraphy or scan or radiolabel* white blood cells or radiolabel* leucocytes or leukoscan or sulesomab or scintimun or besilesomab or BW250 or NCA90 or NCA95 or HMPAO or oxine

O: diagnostic accuracy

(((prosthetic or prosthesis or joint infection) AND (FDG or FDG-PET or PET-CT or fluorodeoxyglucose or positron emission tomography) AND (wbc or scintigraphy or scan or radiolabel* white blood cells or radiolabel* leucocytes or leukoscan or sulesomab or scintimun or besilesomab or BW250 or NCA90 or NCA95 or HMPAO or oxine) AND (diagnostic accuracy AND full text[sb])) AND ("2000/01/01"[PDat]: "2016/12/31"[PDat]) AND Humans[Mesh])))

All three together: 15 papers

Included paper sfor thorough reading: 5 + 4 papers added later

Included paper safter thorough reading: 5

**20.**

**Anti-granulocyte scintigraphy is a good alternative to white blood cells scintigraphy with similar sensitivity and specificity.(mention HAMA, differences between Fab and IgG, repeatability, which one to use in function of pre-test probability etc)**

Level of evidence: 2

P: prosthetic or prosthesis or joint infection

I: leukoscan or sulesomab or scintimun or besilesomab or BW250 or NCA90 or NCA95

C: wbc or scintigraphy or scan or radiolabel* white blood cells or radiolabel* leucocytes or HMPAO or oxine

O: diagnostic accuracy

((((prosthetic or prosthesis or joint infection) AND (((leukoscan or sulesomab or scintimun or besilesomab or BW250 or NCA90 or NCA95)) AND full text[sb] AND ("2000/01/01"[PDat]: "2016/12/31"[PDat]) AND Humans[Mesh])) AND (((wbc or scintigraphy or scan or radiolabel* white blood cells or radiolabel* leucocytes or HMPAO or oxine)) AND full text[sb] AND ("2000/01/01"[PDat]: "2016/12/31"[PDat]) AND Humans[Mesh])) AND (diagnostic accuracy AND full text[sb] AND ("2000/01/01"[PDat]: "2016/12/31"[PDat]) AND Humans[Mesh])))

All three together: 6 papers

Included papersfor thorough reading: 6

Included papersafter thorough reading: 5

**21.**

**Hybrid SPECT-CT imaging can improve localization of infection (and diagnostic accuracy).**

Level of evidence 2

P: prosthetic or prosthesis or joint infection

I: SPECT-CT or SPECT/CT or hybrid imaging

C: -

O: diagnosis of infection or localization accuracy

(((prosthetic or prosthesis or joint infection) AND (SPECT-CT or SPECT/CT or hybrid imaging)) AND (diagnosis of infection or localization accuracy))

All three together: 50 papers

Included papersfor thorough reading: 5

Included papersafter thorough reading: 5

**22.**

**Semi-quantitative analysis of WBC accumulation over time in WBC scan increases diagnostic accuracy for PJI.**

Level of evidence: 3

P: prosthetic joint infection diagnosis OR bone osteomyelitis diagnosis OR pji OR orthopaedic implants infections OR knee OR hip prosthesis osteomyelitis

I: wbcscintigraphy OR wbc scan OR wbc OR radiolabelled white blood cells OR hmpao OR 111In OR radiolabeled leucocytes

C: semiquantitative analysis OR t/b ratio OR target background ratio OR SUV or SPECT/CT

O: diagnosticaccuracy OR osteomyelitis diagnosis

((prosthetic joint infection diagnosis OR bone osteomyelitis diagnosis OR pji OR orthopaedic implants infections OR knee OR hip prosthesis osteomyelitis) AND (wbcscintigraphy OR wbc scan OR wbc OR radiolabelled white blood cells OR hmpao OR 111In OR radiolabeled leucocytes) AND (semiquantitative analysis OR t/b ratio OR target background ratio OR SUV or SPECT/CT) AND (diagnostic accuracy pji OR prosthetic joint infection OR bone osteomyelitis diagnosis OR infection orthopaedic implants OR knee OR hip prosthetic osteomyelitis diagnosis))

All three together: 17

Included papers for thorough reading: 17

Included papers after thorough reading: 5

**23.**

**Combining WBC scan with bone marrow scan increases diagnostic accuracy for PJI detection.**

Level of evidence: 2

P: prosthetic joint infection or bone osteomyelitis or pji or orthopaedic implants or knee or hip prosth*

I: wbc or (radiolabel* and white and blood and cells) or hmpao or oxine or 111In- or 99mTc or technetium-99m or indium-111

C: (bone and marrow and scintigraphy) or colloid* or sulfur or nanocolloid

O: (diagnosis or (diagnostic and accuracy))

(((prosthetic joint infection or bone osteomyelitis or pji or orthopaedic implants or knee or hip prosth*) AND (wbc or radiolabel* and white and blood and cells or hmpao or oxine or 111In- or 99mTc or technetium-99m or indium-111) AND (bone and marrow and scintigraphy or colloid* or sulfur or nanocolloid) AND (Humans[Mesh]) AND ((diagnosis or (diagnostic and accuracy))) AND Humans[Mesh]))

All three together: 164

Included papers for thorough reading: 32

Included papers after thorough reading: 11

**24.**

**MRI is fully feasible in patients with suspicion of PJI.**

Evidence level: 2

P: hip OR knee OR joint AND implant OR prosthesis OR arthroplasty AND infection

I: magnetic resonance imaging

C: -

O: -

(((hip OR knee OR joint) AND (implant OR prosthesis OR arthroplasty) AND (infection)) AND (magnetic resonance imaging))

Papers found: 187

Included papers for thorough reading: 16

Papers selected: 3

**25.**

**MRI has high diagnostic performance in detecting PJI when clinically suspected with no ionizing radiations.**

Evidence level: 2

P: hip OR knee OR joint AND implant OR prosthesis OR arthroplasty AND infection

I: magnetic resonance imaging

C: -

O: diagnostic performance OR diagnosis OR accuracy

((((hip OR knee OR joint) AND (implant OR prosthesis OR arthroplasty) AND (infection)) AND (magnetic resonance imaging)) AND (diagnostic performance OR diagnosis OR accuracy))

Papers found: 174

Included papers for thorough reading: 7

Papers selected: 3
